# Peripheral Neutrophils-Derived Matrix Metallopeptidase-9 Induces Postoperative Cognitive Dysfunction in Aged Mice

**DOI:** 10.3389/fnagi.2022.683295

**Published:** 2022-02-22

**Authors:** Lili Huang, Weitian Tian, Xuemei Chen, Huan Xu, Wanbing Dai, Yizhe Zhang, Xiaodan Wu, Weifeng Yu, Jie Tian, Diansan Su

**Affiliations:** ^1^Department of Anesthesiology, School of Medicine, Renji Hospital, Shanghai Jiao Tong University, Shanghai, China; ^2^Department of Anesthesiology, Shanghai Pulmonary Hospital, Tongji University, Shanghai, China; ^3^Department of Anesthesiology, Shengli Clinical Medical College, Fujian Provincial Hospital, Fujian Medical University, Fuzhou, China

**Keywords:** matrix metallopeptidase- 9, neutrophils, postoperative cognitive dysfunction, anesthesia and surgery, blood–brain barrier

## Abstract

**Background:**

Aging is one of the most important risk factors of postoperative cognitive dysfunction (POCD); however, the mechanisms are still not completely understood. In this study, we explore the roles of matrix metalloproteinase-9 (MMP-9) in aged mice with POCD.

**Methods:**

Appendectomy was performed in 18-month-old C57BL/6 and MMP-9^–/–^ mice under anesthesia to establish the POCD model. Learning and memory were assessed using the Morris water maze (MWM) or Barnes maze. Protein expression of MMP-9 was measured by Western blotting or enzyme-linked immunosorbent assay (ELISA). To explore the role of neutrophils-derived MMP-9 in POCD, we treated mice with anti-Gr-1 monoclonal antibody to deplete peripheral neutrophils. And the percentage of neutrophils and other leukocytes were detected by flow cytometry. We further used sodium fluorescein (NaFlu) to evaluate the blood–brain barrier (BBB) permeability.

**Results:**

The spatial learning and memory ability was injured, and expression of MMP-9 increased in both plasma and the hippocampus after anesthesia/surgery. However, cognitive dysfunction was alleviated in both MMP-9^–/–^ and peripheral neutrophils-depleted mice. The permeability of BBB was increased after anesthesia/surgery while recused by anti-Gr-1 antibody administration.

**Conclusion:**

These findings suggest that peripheral neutrophils-derived MMP-9 could lead to POCD of aged mice through increasing the BBB permeability.

## Introduction

Postoperative cognitive dysfunction (POCD) is a common central nervous system complication after anesthesia and surgery. It increases morbidity and mortality and results in a rise in the premature departure from the workforce (Steinmetz et al., 2009). According to the literature, the occurrence of POCD varies widely from 9 to 60%, resulting from differences in study populations and protocols used to detect POCD (Moller et al., 1998; Newman et al., 2001). Although aging is considered the only definite risk factor of POCD (Rosczyk et al., 2008; Steinmetz and Rasmussen, 2016; Zhong et al., 2020), the reason why elderly patients are more vulnerable to memory deficits after surgery remains ambiguous.

MMP-9 is a member of the MMP family and secreted mostly as an inactive proenzyme, followed by activation via propeptide removal (Klein and Bischoff, 2011; Bchir et al., 2017). MMP-9 can degrade many kinds of proteins, including extracellular matrix, tight junction proteins, and adhesion molecules (Sternlicht and Werb, 2001). Although several studies indicated that MMP-9 increases in the brain after surgery in aged patients, the origin of the increase remains a mystery (Li et al., 2016; Bi et al., 2017).

Many cells secrete MMP-9, including neurons, microglia, astrocytes, macrophages, and neutrophils (Vafadari et al., 2016). In contrast to other cells, neutrophils produce MMP-9 in a particular form, and MMP-9 is stored in neutrophils in the form of complexes, monomers, and oligomers as proenzymes in granules (Masure et al., 1991; Borregaard, 2010). However, unlike other cells, neutrophils do not produce the high-affinity endogenous inhibitor of MMP-9-tissue inhibitors of metalloproteinases (TIMP-1), which makes them a critical source of MMP-9 (Opdenakker et al., 2001; Rowe and Weiss, 2008). Neutrophils are first-line defense leukocytes and increase robustly after the surgical process. And MMP-9 stored in neutrophils can be released quickly after stimulation (Romeo et al., 2002; Vandooren et al., 2013). As a vital barrier between peripheral blood and nervous system, BBB is essential in protecting neural cells from harmful factors in circulation, and accumulating evidence showed that BBB breakdown can contribute to cognitive dysfunction in several neurodegenerative disease, such as Alzheimer’s Disease (AD) (Erickson and Banks, 2013; Montagne et al., 2015). While MMP-9 has been proved to destroy BBB by degrading the extracellular matrix and breaking tight junctions (Bell et al., 2012).

Thus, we hypothesized that increased MMP-9 derived from peripheral neutrophils after anesthesia/surgery leads to learning memory impairment of aged mice by breaking down BBB.

## Materials and Methods

### Ethics

The study was approved by the Animal Care and Use Committee of Shanghai Jiao Tong University, School of Medicine. Treatment of all animals conformed to institutional and National Institutes of Health guidelines. Every effort was made to minimize animal suffering and to reduce the overall number of animals used.

### Animals

Eighteen-month-old male C57BL/6 mice were provided by Sino-British SIPPR/BK Lab (Shanghai, China). The 18-month-old wild-type (WT) and MMP9^–/–^ mice with a FVB genetic background were purchased from Nanjing Biomedical Research Institute of Nanjing University. All animals were housed in standard cages under controlled laboratory conditions (temperature of 22 ± 2°C, 12-h light/12-h dark cycle) with free access to regular rodent pellets and water. All mice were allowed to adapt to their new environment for 7 days before the experiments were initiated. The group size was 8–15 mice for the behavioral tests and 3–6 mice for the molecular biology experiments.

### Anesthesia and Surgery

Appendectomy was performed after neuroleptic anesthesia (intraperitoneal injection of 200 μg/kg fentanyl and 10 mg/kg droperidol), as previously described (Zhao et al., 2016). Previous research reported that this regimen could provide sufficient sedation and pain relief for rodents during surgery without affecting their learning or memory ability (Wan et al., 2007). Mice underwent a standard surgical procedure for appendectomy, in accordance with the procedures reported in a previous study (Xu et al., 2019). First, a small incision of approximately 1 cm was made in the middle abdominal quadrant, followed by immobilization and isolation of the end of the cecum and appendix. Two ligatures were placed proximal to the border of the appendix and the cecum, and division of the appendix was conducted between the two ligatures. To ensure the smoothness and function of the intestine, the remaining appendix was at least two-thirds of the original length. The cecal stump and abdominal cavity were flushed with saline and the intestine returned. Finally, the two-layer abdominal wall was closed. At the end of the surgery, a single dose of butorphanol (0.4 mg/kg, subcutaneously) was administered for postoperative analgesia. Aseptic techniques were used during the entire procedure, and mice recovered from anesthesia within 20 min. A warming system was used throughout the experiment to maintain the temperature of the mice. SpO_2_ was monitored, and no hypoxia (SpO_2_ < 90%) was observed during the procedure.

### *In vivo* Neutrophil Depletion

Based on previous studies, each mouse was injected intraperitoneally with 250 and 100 μg of anti-Gr-1 monoclonal antibody (mAb; clone RB6-8C5; #BE0075, Bio-X-Cell, Lebanon, United States) at 24 and 4 h before the appendectomy, respectively (Liu et al., 2006; Stirling et al., 2009). The RB6-8C5 clone can bind to the myeloid differentiation antigen Gr-1, which is a member of the Ly6 gene family. Previous studies confirmed that the antibody can deplete neutrophils in both blood and spleen for up to 2–3 days after injection (Conlan and North, 1994). Therefore, we administered an additional injection of the antibody (100 μg per mouse) every 2 days until the end of the water maze. Control group mice received treatment with isotype-matched control antibodies (IgG2b isotype control; #BE0089, Bio-X-Cell, Lebanon, United States) at an equivalent dose and on the same schedule.

### Morris Water Maze

To measure the learning and memory ability of mice, Morris Water Maze (MWM) trials were performed on the third day after the appendectomy in accordance with the procedures of our previous studies (Zhao et al., 2016; Xu et al., 2019). The water maze consisted of a circular pool (110-cm diameter and 30-cm high) in which mice were trained to escape from the water by swimming to a hidden platform (1.0 cm beneath the surface of the water). The pool was situated in a room with visual cues, and the position of cues remained unchanged throughout the task. Water was kept at 24 ± 2°C and opacified with dye for all training and testing. The pool was divided into the following four quadrants: target, opposite, adjacent 1, and adjacent 2. The experiments were recorded using a camera connected to a video recorder and a computerized tracking system (Shanghai Jiliang Software Technology Co., Ltd., China).

MWM trials consist of positioning navigation and spatial probe two parts. The positioning navigation test lasted for 4 days, with each mouse receiving four training sessions per day to evaluate spatial learning ability. In each session, the mouse was placed sequentially into water from four different points. Once the mouse located the platform, the trial was terminated, and the mouse was allowed to stay on the platform for 15 s. If the mouse failed to find the platform within 60 s, it was gently guided to the platform and allowed to remain on the platform for 15 s. Four trials were conducted per day, with separated intervals of 5 min. The amount of time spent finding and climbing the platform (escape latency) as well as the swimming speed were recorded by software.

The spatial probe test was performed on the first day after the reference memory test (day 7). In this test, the platform was removed, and the animals were allowed to explore freely for 60 s. We recorded the time spent in the target and opposite quadrant.

### Barnes Maze

The Barnes maze is another method to test the learning and memory ability of mice, which is especially suitable for aged and gene-knocked mice (Han et al., 2012). The maze is a circular platform with 20 holes and a black escape box (target box; 15 × 7 × 7 cm) was placed under one hole. Spatial cues with distinct patterns and shapes were placed on the wall of the testing room. During the trial, 80 dB aversive noise and 500-lux light was turned on to encourage mice to find the target box.

The training stage lasted for 4 days. Four trials were performed each day with an intertrial interval of 20 min. Each mouse’s maximum exploration time was 3 min. During this time, if the animal still could not locate the target, the animal was gently removed from the maze and guided to the target box, where it was allowed to stay 1 min. A spatial probe test was performed on day 5. The target box was removed, which allowed the mice to explore freely for 90 s. A video camera mounted above the platform was used to track the mice; the escape latency, number of correct times, and the number of errors were recorded and calculated using Barnes maze software (Shanghai XinRuan Information Technology Co., Ltd., China).

### Western Blotting

Animals were sacrificed by decapitation, as described in our previous studies (Zhang et al., 2018; Xu et al., 2019). After transcardial perfusion with saline, the hippocampus was quickly dissected on ice, frozen in liquid nitrogen, and stored at −80°C. Hippocampus tissues were homogenized in cold radioimmunoprecipitation assay buffer (Beyotime Biotechnology, China), and the quantity of protein in the supernatants was determined using a bicinchoninic acid protein assay kit (Thermo Fisher Scientific, Waltham, MA, United States). The proteins were separated on 8 or 10% polyacrylamide gels and transferred onto polyvinylidene fluoride membranes (pore size: 0.45 μm; Millipore, Burlington, MA, United States). The membranes were blocked for 1 h at room temperature with 5% non-fat dry milk in TBS/0.1% Tween-20 and then incubated with primary antibody (MMP-9, 1:1,000, Abcam, #ab38898; and actin, 1:10,000, Cell Signaling Technology, #8457, Danvers, MA, United States) overnight at 4°C. The membranes were washed three times in TBS/0.1% Tween-20 for 10 min and then incubated for 2 h at room temperature with appropriate secondary antibodies. The immunocomplexes were visualized using a chemiluminescence peroxidase substrate (Clarity Max Western ECL Substrate), and we used the ChemiDoc XRS system (Bio-Rad, Hercules, CA, United States) to detect the band intensities.

### Enzyme-Linked Immunosorbent Assay

The mice were sacrificed separately at 6, 12, 24, 48, and 72 h after surgery, and blood samples were immediately extracted through the heart. Blood samples were centrifuged for 15 min at 1,000 × *g* within 30 min of collection. To completely remove the platelets, we performed an additional centrifugation step of the plasma at 10,000 × *g* for 10 min at 2–8°C. The plasma was removed and stored at −80°C for further processing. MMP-9 concentrations in plasma were determined using a commercially available quantitative enzyme-linked immunosorbent assay (ELISA) kit (R&D Systems, #MMPT90, United States), according to the recommendations of the manufacturer. Each experimental condition was tested in three different wells and measured in duplicate. The optical density was measured with a microplate reader (Varioskan Flash 3001, Thermo Fisher Scientific, Finland) at a wavelength of 450 nm.

### Flow Cytometry

Single-cell suspensions were prepared from the blood and brain tissues of control or surgery group mice at postoperative day 2. Brain tissues were dissociated by MACS Neural Tissue Dissociation Kit (Miltenyi Biotec GmbH, Teterow, Germany) according to the protocol of the manufacturer. The cells were incubated with Fixable Viability Stain 510 (BD PharMingen, San Diego, CA, United States) for 15 min at room temperature protected from light. After that, these cells were stained with fluorochrome-conjugated antibodies. The following mouse antibodies were used in this study: anti-CD45 (clone 104, #560694, BD Biosciences, San Diego, United States), anti-CD11b (clone M1/70, #561098, BD Biosciences, San Diego, United States), anti-Ly6G (clone 1A8, #127614, Biolegend, United States), anti-Ly6C (clone HK1.4, #128007, Biolegend, United States), anti-F4/80 (clone T45-2342, #565411, BD Biosciences, San Diego, United States), anti-CD3 (clone 145-2C11, #551163, BD Biosciences, San Diego, United States), anti-CD19 (clone 1D3, #561738, BD Biosciences, San Diego, United States), anti-NK1.1 (clone PK136, #45-5941-80, Invitrogen, Carlsbad, United States), and anti-CD11c (clone HL3, #561022, BD Biosciences, San Diego, United States). All acquisitions were performed using BD Biosciences FACSVerse™ and analyzed using FlowJo 10.4 software.

### Evaluation of Blood–Brain Barrier Permeability

BBB permeability was assessed by measuring the brain level of NaFlu tracer (376 Da, 10%, 2 ml/kg, #F6377, Sigma-Aldrich) which was injected through the tail-vein and allowed to circulate for 30–40 min. Then, mice were transcardially perfused with saline for about 15 min and the brain were immediately dissected on the ice. Brain tissues were weighed and homogenized in 0.25 ml phosphatebuffered saline (PBS). An equal volume of 60% trichloroacetic acid was added to the tissue homogenate and mixed with a vortex for 2 min to precipitate proteins. Samples were later cooled at 4°C for 30 min and centrifuged at 12,000 g for 20 min. The concentration of tracer in supernatant was measured at excitation wavelength of 460 nm and emission wavelength of 515 nm using a spectrophotofluorometer. NaFlu was expressed as μg/g of brain tissue against a standard curve.

### Immunohistochemistry

Animals were sacrificed and perfused intracardially with saline followed by 4% paraformaldehyde in 0.1 M phosphate buffer (PB, pH 7.4). Brains were then removed and postfixed in 4% paraformaldehyde overnight, then we embedded them with paraffin. The paraffin sections were dried for 1 h at 60°C and dewaxed with xylene. After a graded series of ethanol solutions, antigen repair, incubation with 3% H_2_O_2_, and blocking (5% BSA), slides were incubated with mouse anti-TMEM119 (Abcam, #ab209064, United Kingdom) overnight at 4°C, then rinsed with PBS and incubated with secondary antibody (HRP labeled) for 50 min at room temperature. We visualized them with diaminobenzidine. Images were acquired with a Leica TCS SP2 confocal laser scanning microscope. Image J was used for the quantification of the positive percentage. The mean integral optical density (IOD) was calculated in 3 fields of the hippocampus for each slide.

### Statistics

All statistical analyses were performed using SPSS 20.0. Results were expressed as mean ± SEM, and statistical significance was set at *P* < 0.05. A normality test was conducted before analysis and all data were normally distributed. Statistically significant differences between two groups were determined by Student *t*-test. For three or more groups, statistical analysis was performed using one-way analysis of variance (ANOVA). We used two-way ANOVA with repeated measures to analyze the water maze and Barnes maze escape latency and average speed. One-way ANOVA was used for the probe quadrant trial data. The data obtained by ELISA and Western blot analysis were analyzed by ANOVA, followed by Bonferroni *post hoc* analysis.

## Results

### Anesthesia/Surgery–Induced Learning and Memory Impairment and Increased Matrix Metalloproteinase-9 in Aged Mice

**FIGURE 1:**
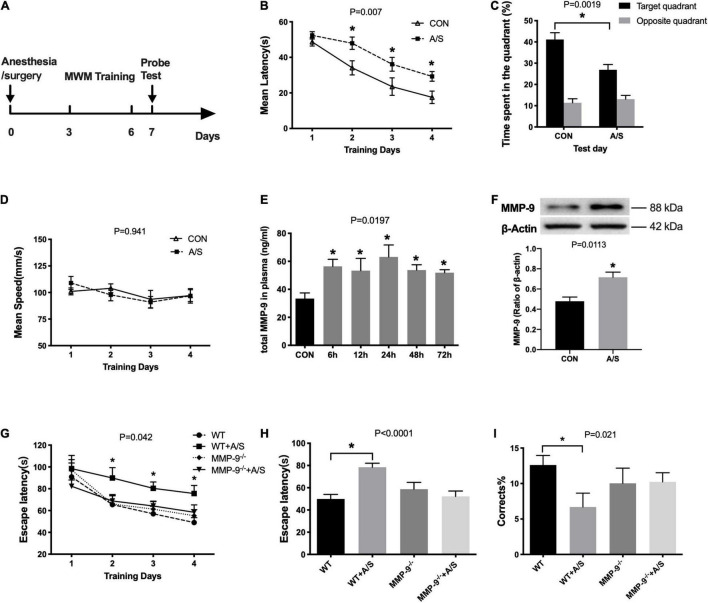
Anesthesia/surgery–induced learning and memory dysfunction and increased MMP-9 in aged mice. **(A)** Schematic timeline of the MWM experimental paradigm. **(B)** The mean escape latency in the anesthesia/surgery (A/S) group increased significantly on the second, third, and fourth days when compared with that in the control (CON) group during training days, *F*_(1_, _22)_ = 8.808, *P* = 0.007. **(C)** In the anesthesia/surgery group, the time spent in the target quadrant was shorter than in the control group in the probe test section, *P* = 0.0019. **(D)** There was no difference between the two groups in the average swimming speed, *F*_(1_, _22)_ = 0.674, *P* = 0.941. (*n* = 12 per group in water maze). **(E)** Total MMP-9 in plasma was increased as early as 6 h after anesthesia/surgery until 72 h, *F*_(5_, _31)_= 3.181, *P* = 0.0197. **(F)** Anesthesia/surgery significantly increased the MMP-9 levels in the hippocampus as compared with the control group, *P* = 0.0113. (*n* = 4–7 per group). **(G)** The escape latency of wild-type mice after anesthesia/surgery (WT + A/S) was remarkably longer than that of wild-type mice in the control group (WT), whereas there was no significance change in the MMP-9^–/–^ mice between the control (MMP-9^–/–^) and anesthesia/surgery group (MMP-9^–/–^ + A/S) during the training days, *F*_(3_, _42)_ = 2.978, *P* = 0.042. **(H,I)** In the Barnes maze test trial, mice in the WT + A/S group showed a longer escape latency **(H)** and had fewer correct times **(I)** to find the escaping box, whereas mice in the MMP-9^–/–^ + A/S group had a performance comparable with that of mice without surgery, **(H)**, *P* < 0.0001; **(I)**, *P* = 0.021. (*n* = 8–15 per group in Barnes maze). Data are expressed as means ± SE. *P < 0.05 compared with the control or WT control group.

Previous studies have shown that anesthesia/surgery impairs learning and memory in aged mice. Thus, we used MWM here to evaluate reference memory after anesthesia/surgery (Figure 1A). Results showed that the escape latency of mice after surgery was significantly prolonged on the second, third, and fourth days when compared with that of the control group (Figure 1B). In the probe test, there was an obvious decrease in the percentage of swimming time spent in the target quadrant in the anesthesia/surgery group (Figure 1C). In addition, their swimming ability was not affected by anesthesia/surgery, as no difference was found in the average swimming speed between each group (Figure 1D).

To explore the changes of MMP-9 in the occurrence of POCD, we used ELISA to measure the plasma levels of total MMP-9 at different time points after anesthesia/surgery. The results showed that MMP-9 was elevated promptly at 6 h and lasted more than 72 h (Figure 1E). We also found that the active MMP-9 protein level was elevated in the hippocampus in the anesthesia/surgery group (Figure 1F).

### Matrix Metalloproteinase-9 Deficiency Rescued Learning and Memory Impairment After Anesthesia/Surgery

Because MMP-9 obviously increased after anesthesia/surgery in aged mice, we used 18-month-old MMP-9^–/–^ mice to further confirm the role that MMP-9 plays in the occurrence of POCD. We performed a Barnes maze test to assess the spatial learning and memory ability of mice, in which mice learned to rapidly escape a brightly lit circular field by finding a specific dark escape hole at its periphery. We found that wild-type mice failed to improve their learning performance during the first 4 days of training (Figure 1G) and had substantially declined in the probe trials on the fifth day after anesthesia/surgery (Figures 1H,I), whereas MMP-9^–/–^ mice that underwent appendectomy did not show cognitive function impairment, because they performed comparably well with MMP-9^–/–^ mice without surgery [Figures 1G–I; G, *F*_(3_, _42)_ =2.978, *P* = 0.042; H, *P* < 0.0001; I, *P* = 0.021].

### Neutrophils in Peripheral Blood but Not Brain Increased Significantly After Anesthesia/Surgery

**FIGURE 2:**
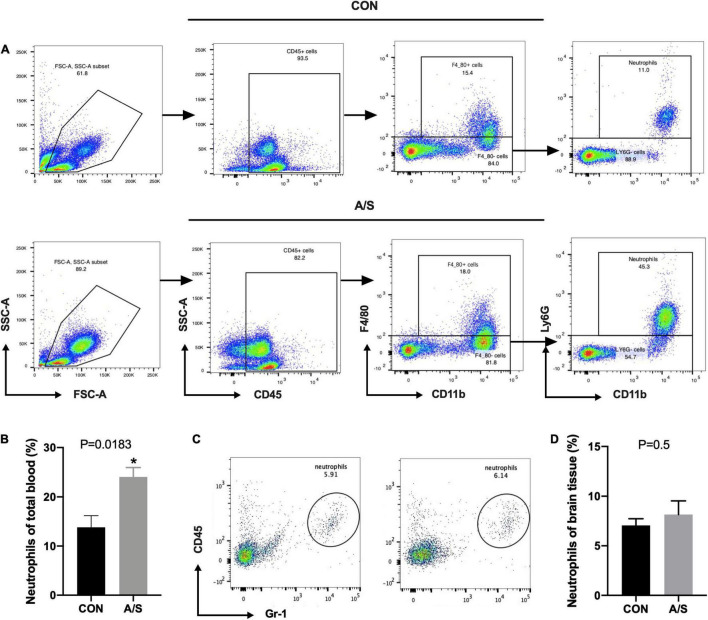
Neutrophils in peripheral blood but not brain increased significantly after anesthesia/surgery. **(A)** Representative flow cytometry plots show the gating strategy of neutrophils (CD45^+^ CD11b^+^ F4/80^–^ Ly6G^+^) in peripheral blood. **(B)** The percentage of neutrophils increased significantly 24 h after anesthesia/surgery, *P* = 0.0183. **(C,D)** There was no significance of neutrophils proportion in brain between CON and A/S groups, *P* = 0.5. Data are expressed as mean ± SE (*n* = 3–4 per group). *P < 0.05 compared with that of the control group.

**FIGURE 3:**
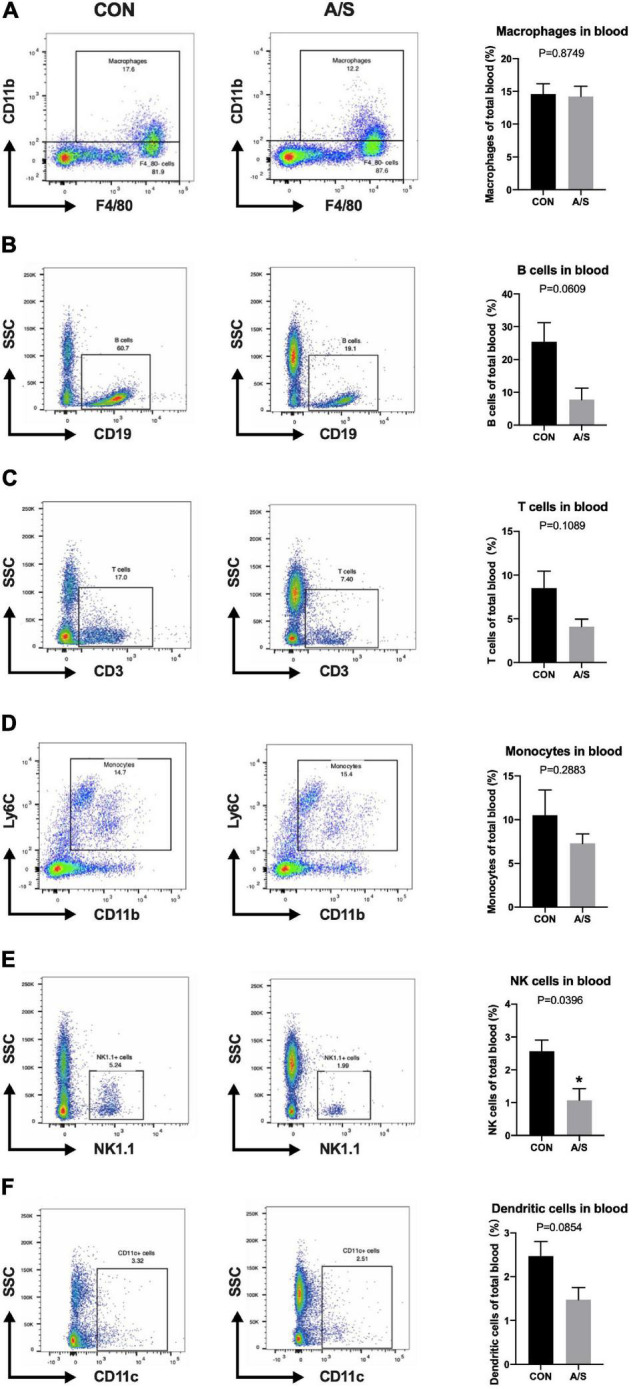
The proportions of leukocytes other than neutrophils were not increased after anesthesia/surgery. Representative flow cytometry data of blood showed the percentage of macrophages (CD45^+^ CD11b^+^ F4/80^+^), B cells (CD45^+^ CD19^+^), T cells (CD45^+^ CD3^+^), monocytes (CD45^+^ CD11b^+^ F4/80^–^ Ly6G^–^ Ly6C^+^), NK cells (CD45^+^ NK1.1^+^) and dendritic cells (CD45^+^ CD11c^+^) at 24 h after anesthesia/surgery. The percentage of NK cells (**E**, *P* = 0.0396) were relatively decreased because of the increase of neutrophils after anesthesia/surgery compared with control group, while no significant difference was found in the percentage of macrophages (**A**, *P* = 0.8749), B cells (**B**, *P* = 0.0609), T cells (**C**, *P* = 0.1089), monocytes (**D**, *P* = 0.2883), and dendritic cells (**F**, *P* = 0.0854). (*n* = 3–4 per group). Data are expressed as means ± SE. *P < 0.05 compared with that of the control group.

Since MMP-9 plays an important role in learning and memory impairment after anesthesia/surgery, we questioned where the increased MMP-9 came from. Neutrophils account for the majority of leukocytes—much more than monocytes and lymphocytes—and secrete MMP-9 in distinct way from other cells. Therefore, we determined the proportions of neutrophils and other leukocyte types in blood using flow cytometry. The results showed that the percentage of neutrophils (CD45^+^ CD11b^+^ F4/80^–^ Ly6G^+^) increased significantly after anesthesia/surgery compared with control group (Figures 2A,B), whereas the percentages of other leukocyte types, including macrophages, monocytes, T cells, and B cells, showed no obvious change (Figure 3). Interestingly, the difference of neutrophils in brain tissue was comparable between each group (Figures 2C,D), which indicated that it was peripheral but not central neutrophils played important roles in anesthesia/surgery induced learning memory impairment.

### Peripheral Neutrophils Are the Primary Source of Increased Matrix Metalloproteinase-9 After Anesthesia/Surgery

**FIGURE 4:**
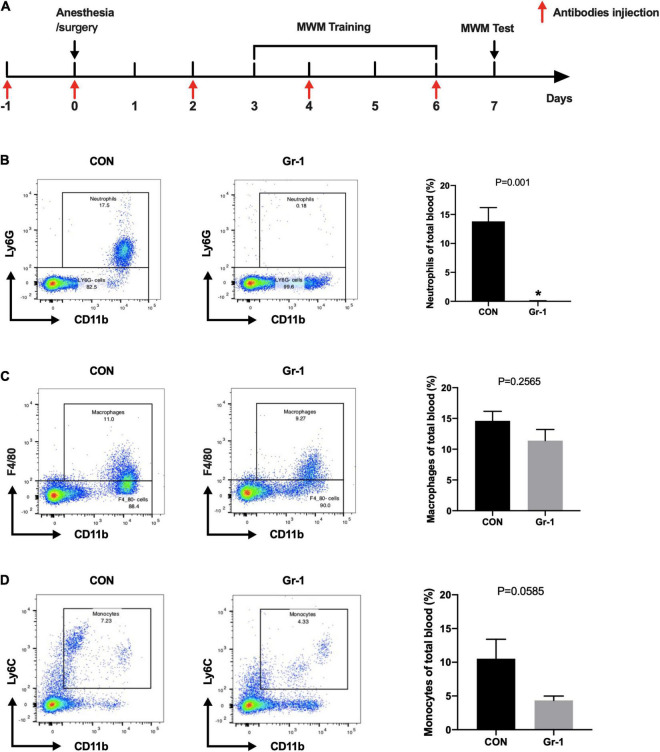
Efficiency of neutrophils depletion with anti-Gr-1 antibody. **(A)** Schematic timeline of the MWM experimental paradigm with neutrophils depletion treatment. **(B)** After anti-Gr-1 treatment, circulating neutrophils were effectively depleted. The percentage of neutrophils in whole blood was calculated, and the efficiency of the depletion is shown (*n* = 3–4 per group, *P* = 0.001). Proportions of macrophages (**C**, *P* = 0.2565) and monocytes (**D**, *P* = 0.0585) were not decreased significantly after anti-Gr-1 administration. Data are expressed as mean ± SE (*n* = 3–4 per group). *P < 0.05 compared with that of the control group.

Neutrophils greatly increase after surgery and are also known to release abundant MMP-9. Thus, we treated the mice with anti-Gr-1 monoclonal antibody (RB6-8C5) intraperitoneally to deplete the peripheral blood neutrophils before commencing anesthesia/surgery (Figure 4A). As the figure showed, neutrophils in blood were mostly significantly eliminated (Figure 4B). Since anti-Gr-1 antibody may also affect the other myeloid cells, we assessed the proportions of macrophages and monocytes in whole blood. Results showed that the percentages of monocytes and macrophages had no statistically significant difference after neutrophils depletion by anti-GR-1 antibody (Figures 4C,D). To exclude the possibility that anti-Gr-1 antibody administration produce an effect on the microglia, which are considered as the resident macrophages of nervous system, we also performed immunohistochemistry staining on hippocampus, while no obvious change was found in microglia marked by TMEM119 (Supplementary Figure 1).

**FIGURE 5:**
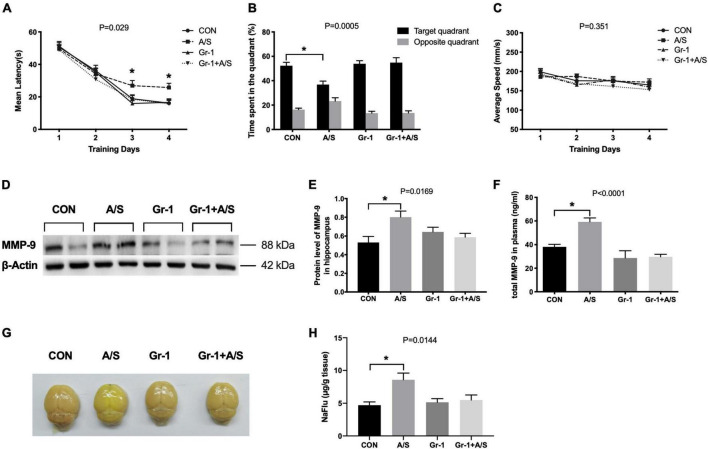
Peripheral Gr-1^+^ neutrophils depletion alleviated anesthesia/surgery–induced learning and memory impairment and BBB disruption in aged mice. **(A–C)** In the MWM, aged mice had a profound deficit in spatial learning and memory ability after undergoing anesthesia/surgery. These mice showed an increased maze escape latency during the 4 days of repeated training trials **(A)** and a shorter time spent in the target quadrant in the probe test section **(B)**. In contrast, mice with Gr-1^+^ neutrophil depletion (Gr-1 + A/S) showed improvement in performance for finding the platform and stayed longer in the target area **(A,B)**. In addition, there was no difference in the average swimming speed among these four groups **(C)**, **(A)**, *F*_(3_, _44)_ = 3.293, *P* = 0.029; **(B)**, *P* = 0.0005; **(C)**, *F*_(3_, _44)_ = 1.122, *P* = 0.351 (*n* = 12 per group). **(D,E)** Depletion of peripheral Gr-1^+^ neutrophils alleviated the elevation of MMP-9 induced by anesthesia/surgery in the hippocampus, *P* = 0.0169. **(F)** Compared with the control group, the total MMP-9 concentration in the plasma of the anesthesia/surgery group showed a remarkable increase at 24 h after anesthesia/surgery; by contrast, there was no difference after anti-Gr-1 treatment between the Gr-1 + A/S group and Gr-1 group, *P* < 0.0001. **(G)** Representative whole brains showed sodium fluorescein (NaFlu) extravasation to evaluate the BBB permeability at 24 h after anesthesia/surgery. **(H)** The statistical data of NaFlu in brain tissue showed the permeability of BBB increased after anesthesia/surgery but there was no significant difference between Gr-1 and Gr-1 + A/S group, *P* = 0.0144. Data are expressed as means ± SE (*n* = 4–6 per group). *P < 0.05 compared with that of the control group.

The MWM results showed that 18-month-old mice exhibited spatial learning and memory ability deficits after anesthesia/surgery, with a longer escape latency and shorter time spent in the target quadrant (Figures 5A,B). However, after depleting the peripheral neutrophils, impairment in learning and memory after anesthesia/surgery was significantly improved (Figures 5A–C). The increasement of MMP-9 in the plasma and hippocampus after anesthesia/surgery was also alleviated in mice with neutrophils depletion (Figures 5D–F). Therefore, these results indicated that neutrophils might be the major source of MMP-9, and the depletion of peripheral neutrophils could effectively diminish the impairment of learning and memory induced by the increasement of MMP-9 after anesthesia/surgery.

### Depletion of Neutrophils Alleviated the Increasing Blood–Brain Barrier Permeability Induced by Anesthesia/surgery

To elucidate whether MMP-9 in peripheral blood translocated into the brain and delivered its degradation effect, we used the sodium fluorescein (NaFlu) as a tracer to evaluate BBB permeability quantitatively. Results showed BBB permeability was increased after anesthesia/surgery. However, when we treated the aging mice with anti-Gr-1 antibody, BBB still kept low permeability even with identical anesthesia/surgery (Figures 5G,H).

## Discussion

Our present study demonstrated that MMP-9 protein levels in both plasma and the hippocampus were obviously increased after anesthesia/surgery in aged mice. MMP-9 deficiency and neutrophils depletion rescued the cognitive dysfunction, and BBB disruption was also alleviated after the treatment of anti-Gr-1 antibody.

Over the past few years, emerging evidence has pointed toward a significant role of MMP-9 in cognitive dysfunction. van der Kooij et al. (2014) demonstrated that because increased MMP-9 promoted nectin-3 cleavage in the hippocampus, MMP-9 was responsible for stress-induced chronic social and cognitive decline. Similarly, clinical research also showed that in patients with systemic lupus erythematosus with neuropsychiatric manifestations, especially cognitive dysfunction, significantly elevated levels of MMP-9 were detected in serum, accompanied by brain magnetic resonance imaging abnormalities (Ainiala et al., 2004). However, little is known about MMP-9 in POCD. Consistent with previous studies (Li et al., 2016; Zhang et al., 2016), our results found that MMP-9 was increased after anesthesia/surgery in the hippocampus, and we also revealed a dynamic change of total MMP-9 concentration in plasma. To ultimately determine the role MMP-9 play in POCD, aged MMP-9^–/–^ mice were submitted to surgery and performed the Barnes maze test. We found that wild-type mice in the anesthesia/surgery group took longer to identify the target box than those in the control group, suggesting that surgery and anesthesia induced learning and memory impairment in the wild-type mice. However, anesthesia/surgery did not influence the time that MMP-9^–/–^ mice spent in identifying the target box both in training and test trials.

Although many kinds of cells are capable of secreting MMP-9, neutrophils are reported to be the major source of MMP-9 (Kurihara et al., 2012; Vlahos et al., 2012) and account for 40–75% of leukocytes—which is much more than other cells like monocytes and lymphocytes. Importantly, the way that neutrophils secrete MMP-9 is distinct from other cells, as MMP-9 is stored in neutrophils in the form of complexes, monomers, and oligomers as proenzymes in granules. Since neutrophils do not produce TIMP-1 or gelatinase A, in contrast with monocytes and tumor cells, they are known to be a main source of MMP-9 for biochemical and biological studies (Opdenakker et al., 2001). In the present study, we observed changes in different kinds of leukocytes in peripheral blood and found that only neutrophils sharply increased after anesthesia/surgery. Although anti-Gr-1 can deplete various MMP-9-secreting cells, such as monocytes and macrophages, the proportion of these cells was not increased after anesthesia/surgery. Thus, neutrophils are considered the majority source of MMP-9. Normally, a small number of neutrophils in the central nervous system (CNS) exist in the meninges, pia mater, and cerebrospinal fluid; they are rarely found in the brain parenchyma due to the blood–brain barrier (BBB) (Kanashiro et al., 2020). Accordingly, only a few neutrophils were detected in the brain tissue in our study. Furthermore, there was no significant difference in neutrophils in the brain between the control and A/S groups, suggesting that neutrophils in the periphery did not cross the BBB and that increased MMP-9 in the hippocampus might originate from peripheral blood.

BBB is an important structure that is composed of endothelial cells, pericytes, astrocyte, tight junctions and extracellular matrix to protect the brain from harmful substances circulating in the blood (Zlokovic, 2008). BBB disruption is considered an important mechanism in the occurrence of cognitive decline in cerebral small vessel disease and various neurodegenerative diseases, such as AD and Parkinson’s disease (PD) (Qin et al., 2019; Jia et al., 2020; Wang et al., 2020; Guo et al., 2021). It was proved that BBB breakdown can lead to neuroinflammation and neuronal damage due to the importance of this barrier in the maintenance of CNS homeostasis (Yu et al., 2019). As an important protease of the MMPs family, MMP-9 has been shown to disrupt the BBB via degradation of the capillary basement membrane and tight junction proteins (Bell et al., 2012; Weekman and Wilcock, 2016). Similarly, Bi et al. (2017) showed that BBB disruption and neuroinflammation after surgery eventually resulted in learning memory impairment of aged mice. The results of NaFlu in our study also showed that the BBB permeability was increased after suffering anesthesia/surgery, but anti-GR-1 antibody prevented the increase of permeability. While treating the aging mice with anti-Gr-1 antibody to deplete neutrophils, the cognitive dysfunction and elevated MMP-9 were all rescued. Therefore, we speculated MMP-9 in peripheral blood might translocate into the brain to deliver its degradation effect via increasing BBB permeability.

There are several limitations in our study. To obtain a higher depletion efficiency, we chose anti-Gr1 (RB6-8C5) antibody rather than anti-Ly6G (1A8) antibody in our experiment. Anti-Gr-1 has non-specific targeting while anti-Ly6G has a lower efficiency than anti-Gr-1 to deplete neutrophils (Boivin et al., 2020). However, the RB6-8C5 clone of anti-Gr-1 antibody cannot only bind to Ly6G, but also Ly6C, which is found on a subset of monocytes and lymphocytes. Therefore, we detected the proportions of macrophages and monocytes, and found there was no statistical significance after administration of anti- Gr-1 antibody. As the resident macrophages of brain, microglia might be affected by anti- Gr-1 antibody. However, as a macro-molecular protein, anti-GR-1 antibody is hardly to cross the intact BBB under normal circumstances. And in our study, the anti-Gr-1 antibody was treated at 24 and 4 h before anesthesia/surgery as the BBB was intact. In our immunohistochemistry experiment of microglia in hippocampus, there was no change of microglia density after administration of anti-GR-1 antibody. Therefore, we considered that the anti-GR-1 antibody mainly worked in the periphery and had no adverse effect on microglia.

## Conclusion

In summary, our findings identify a key role of peripheral neutrophils–derived MMP-9 in POCD and highlight MMP-9 as novel target for the treatment of POCD in elderly patients.

## Data Availability Statement

The original contributions presented in the study are included in the article/Supplementary Material, further inquiries can be directed to the corresponding author/s.

## Ethics Statement

The animal study was reviewed and approved by the Animal Care and Use Committee of Renji Hospital, Shanghai Jiao Tong University School of Medicine, Shanghai, China.

## Author Contributions

DS, XC, JT, XW, and WY: study conception. DS, LH, and WT: study design. DS, LH, XC, and WY: study conduct. LH, WT, WD, HX, and DS: data analysis. HX, YZ, and WT: data interpretation. DS, LH, and XC: drafting of the manuscript. DS, WT, HX, YZ, WD, JT, XW, and WY: critical revision of the manuscript for important intellectual content. All authors contributed to the article and approved the submitted version.

## Conflict of Interest

The authors declare that the research was conducted in the absence of any commercial or financial relationships that could be construed as a potential conflict of interest.

## Publisher’s Note

All claims expressed in this article are solely those of the authors and do not necessarily represent those of their affiliated organizations, or those of the publisher, the editors and the reviewers. Any product that may be evaluated in this article, or claim that may be made by its manufacturer, is not guaranteed or endorsed by the publisher.

## Supplementary Material

Supplementary Figure 1 Anti-Gr-1 antibody did not change the density of microglia. **(A)** Representative immunohistochemistry images of TMEM119-positive cells (microglia marker) of hippocampal CA1 section showed anti-Gr-1 treatment did not affect the density of microglia. Scale bar: 50 μm. **(B)** Quantification of the mean integral optical density of TMEM119-positive microglia. *P* = 0.6827. Data are expressed as means ± SE (*n* = 3 per group). **P* < 0.05 compared with that of the control group.

## Abbreviations

POCD: postoperative cognitive dysfunction
MMP-9: matrix metalloproteinase-9
ELISA: enzyme-linked immunosorbent assay
TIMPs: tissue inhibitors of metalloproteinases
PBS: phosphate-buffered saline
MWM: Morris water maze
ANOVA: one-way analysis of variance
CNS: central nervous system
BBB: blood–brain barrier
AD: Alzheimer’s Disease
PD: Parkinson’s disease.
