# Supplementary figures and images for: Peripheral Neutrophils-Derived Matrix Metallopeptidase-9 Induces Postoperative Cognitive Dysfunction in Aged Mice
Source: Front Aging Neurosci. 2022 Feb 22;14:683295. doi: 10.3389/fnagi.2022.683295 (PMC8902411; doi:10.3389/fnagi.2022.683295)

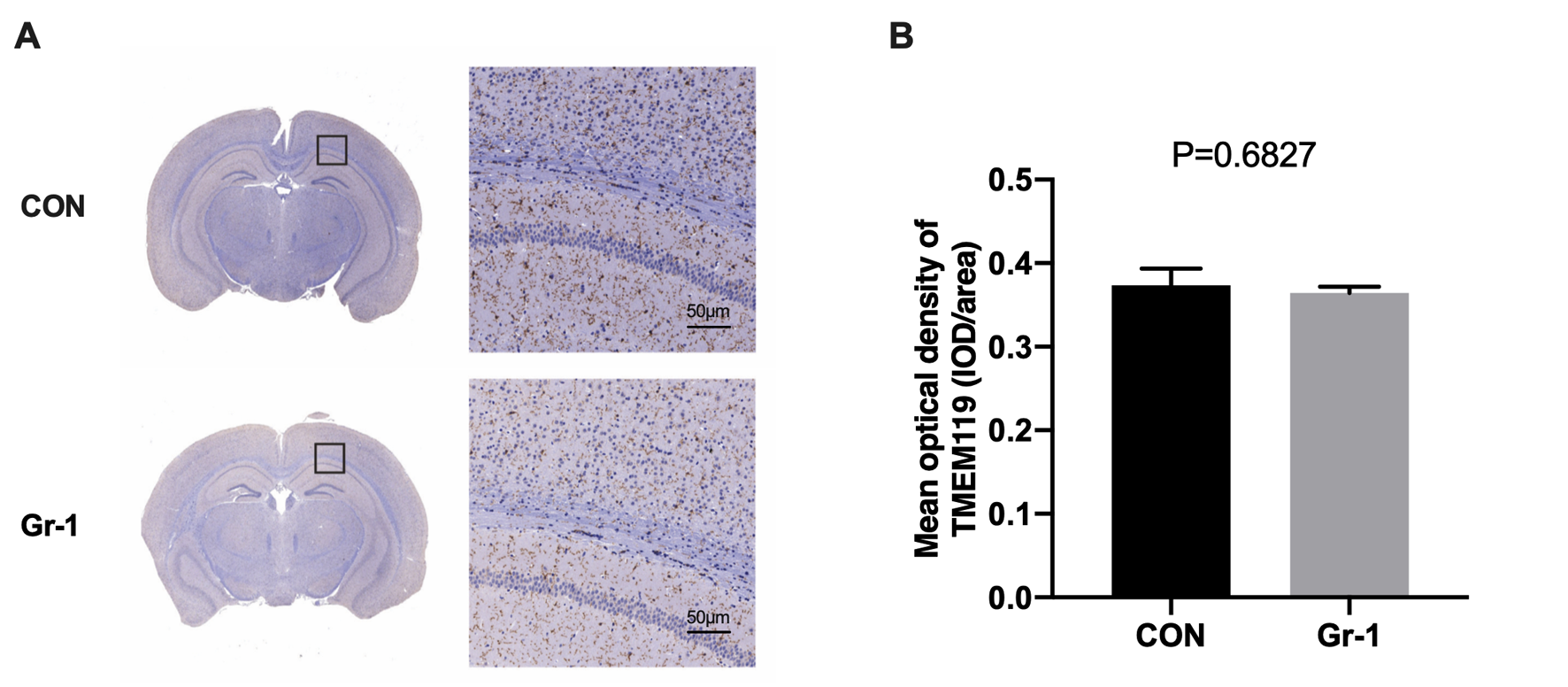

Supplement: Supplementary Figure 1 — Anti-Gr-1 antibody did not change the density of microglia. (A) Representative immunohistochemistry images of TMEM119-positive cells (microglia marker) of hippocampal CA1 section showed anti-Gr-1 treatment did not affect the density of microglia. Scale bar: 50 μm. (B) Quantification of the mean integral optical density of TMEM119-positive microglia. P = 0.6827. Data are expressed as means ± SE (n = 3 per group). *P < 0.05 compared with that of the control group. [file Image_1.TIFF]
